# Multivariate testing and effect size measures for batch effect evaluation in radiomic features

**DOI:** 10.1038/s41598-024-64208-z

**Published:** 2024-06-17

**Authors:** Hannah Horng, Christopher Scott, Stacey Winham, Matthew Jensen, Lauren Pantalone, Walter Mankowski, Karla Kerlikowske, Celine M. Vachon, Despina Kontos, Russell T. Shinohara

**Affiliations:** 1 Department of Bioengineering, University of Pennsylvania, Philadelphia, PA 19104 USA; 2 Department of Radiology, Center for Biomedical Image Computing and Analysis (CBICA), University of Pennsylvania, Philadelphia, PA 19104 USA; 3 Penn Statistics in Imaging Endeavor (PennSIVE), Department of Biostatistics, Epidemiology, and Informatics, University of Pennsylvania, Philadelphia, PA 19104 USA; 4 Mayo Clinic, Rochester, MN 55905 USA; 5 University of California, San Francisco, CA 94121 USA; 6 Center for Innovation in Imaging Biomarkers and Integrated Diagnostics (CIMBID), Columbia University, New York, NY 10027 USA

**Keywords:** Statistics, Prognostic markers

## Abstract

While precision medicine applications of radiomics analysis are promising, differences in image acquisition can cause “batch effects” that reduce reproducibility and affect downstream predictive analyses. Harmonization methods such as ComBat have been developed to correct these effects, but evaluation methods for quantifying batch effects are inconsistent. In this study, we propose the use of the multivariate statistical test PERMANOVA and the Robust Effect Size Index (RESI) to better quantify and characterize batch effects in radiomics data. We evaluate these methods in both simulated and real radiomics features extracted from full-field digital mammography (FFDM) data. PERMANOVA demonstrated higher power than standard univariate statistical testing, and RESI was able to interpretably quantify the effect size of site at extremely large sample sizes. These methods show promise as more powerful and interpretable methods for the detection and quantification of batch effects in radiomics studies.

## Introduction

Radiomics, or the high-throughput extraction of quantitative features from medical images, is an emerging field at the intersection of medicine and data science that enables leveraging standard-of-care images in the prediction of outcomes relevant to precision oncology^1,2^. While the applications of radiomics are promising, multi-center study designs are often needed to demonstrate sufficient statistical power and generalizability for clinical translation^3^. However, multi-center datasets are often heterogeneous in imaging equipment, scan protocols, and post-processing algorithms, resulting in images that are broadly equivalent clinically but contain unwanted variation associated with technical factors, termed “batch effects”^4^. Many studies in radiomics from magnetic resonance imaging (MRI), positron emission tomography (PET), and computed tomography (CT) have shown that differences in acquisition parameters, site, and scanner can reduce feature reproducibility and affect downstream predictive analyses^5–9^.

Increasing awareness of how problematic batch effects are in radiomics studies has increased interest in batch effect correction, or “harmonization” methods, that aim to correct batch effects associated with differences in acquisition and post-processing. Harmonization methods can be applied either before feature extraction by standardizing protocols and image pre-processing or after feature extraction by correcting the feature matrices^10,11^. One of the most popular harmonization methods in radiomics is ComBat, a statistical harmonization tool that uses an empirical Bayes framework to estimate the parameters needed to correct differences in the location and scale of the feature distribution attributable to batch^11–13^. ComBat has been employed in many radiomics analyses, with demonstrated benefits in improving study power and reproducibility^14,15^. The effective implementation of tools for correcting batch effects has resulted in the creation of improved harmonization methods, and a greater number of studies are being carried out to assess their effectiveness across a wider range of datasets^11,16^. Some of these advancements include generalizing ComBat to multiple batch variables and scenarios when batch variables are unknown, as well as application of ComBat to covariance structures and longitudinal data^17–20^. However, the methods used to evaluate when harmonization is necessary, and the performance of harmonization methods are varied and inconsistent.

Quantification of harmonization performance requires standardized and effective metrics that detect and measure batch effects in data. A common method for evaluation in both neuroimaging and radiomics data is statistical testing for differences in distribution attributable to batch^13,21,22^. In this paradigm, when feature distributions are split along batch, the sub-distributions should be identical if no batch effects are present in the data. Radiomics studies have historically used univariate statistical tests such as the Wilcoxon Rank Sum (WRS) test, t-test, Kolmogorov-Smirnov (KS) test, and Anderson-Darling (AD) test to assess for significant differences in distribution associated with batch^14,15,23^. However, tests for differences in location such as the WRS test and t-test may not detect more general differences in distribution. The KS test enables testing for more general differences in distribution, but can only be used for testing between two groups and not when batch variables contain three or more groups. The AD test, which is available for k-sample testing, enables assessment of differences in distribution for three or more groups but is sensitive to cases where the bulk of the distributions are aligned due to the heavier weight it places on the tails of the distribution^18^. These weaknesses indicate that the most commonly used statistical tests for detecting batch effects are inadequate for the complex distributions often observed in radiomic features.

In addition, statistical tests that rely on a p-value become less effective at extremely large sample sizes where the p-value becomes significant at extremely small effect sizes^24^. At such large sample sizes, the p-value becomes uninformative because increases in p-value cannot be interpreted as decreases in effect size. In addition, statistical tests relying on p-values with an arbitrary threshold for significance have also come under increasing criticism by the statistics community in recent years because the p-value does not quantify effect size or provide information regarding result reproducibility^25^.

In this study, we adapted the multivariate statistical test Permutational Analysis of Variance (PERMANOVA) to overcome the limitations of standard univariate testing by modeling complex nonlinear relationships between batch variables and features, without making any distributional assumptions. We also investigated the use of the Robust Effect Size Index (RESI) as a metric to quantify the effect size of batch and better characterize the magnitude of batch effects. In Section 2, we describe the PERMANOVA and RESI methods, as well as the simulation model and real imaging data used for evaluating their performance. In Section 3, we describe our findings regarding the batch effect evaluation performance of the proposed metrics relative to traditional statistical tests in real and simulated data. In Section 4, we discuss the results of power analysis for PERMANOVA and the interpretation of RESI in the context of batch effect detection. By utilizing these methods, we aim to achieve better statistical power and more interpretable quantification of batch effects in radiomics data. These techniques can be incorporated into a unified batch effect assessment pipeline, where PERMANOVA identifies batch effects at the dataset level, while RESI quantifies the magnitude of batch effects at the feature level.

These methods can be integrated into a single batch effect evaluation pipeline, where PERMANOVA is used to screen the data for batch effects at the dataset-level and the RESI is used to quantify the effect size of batch at the feature-level.

## Results

### Simulated data

**Figure 1:**
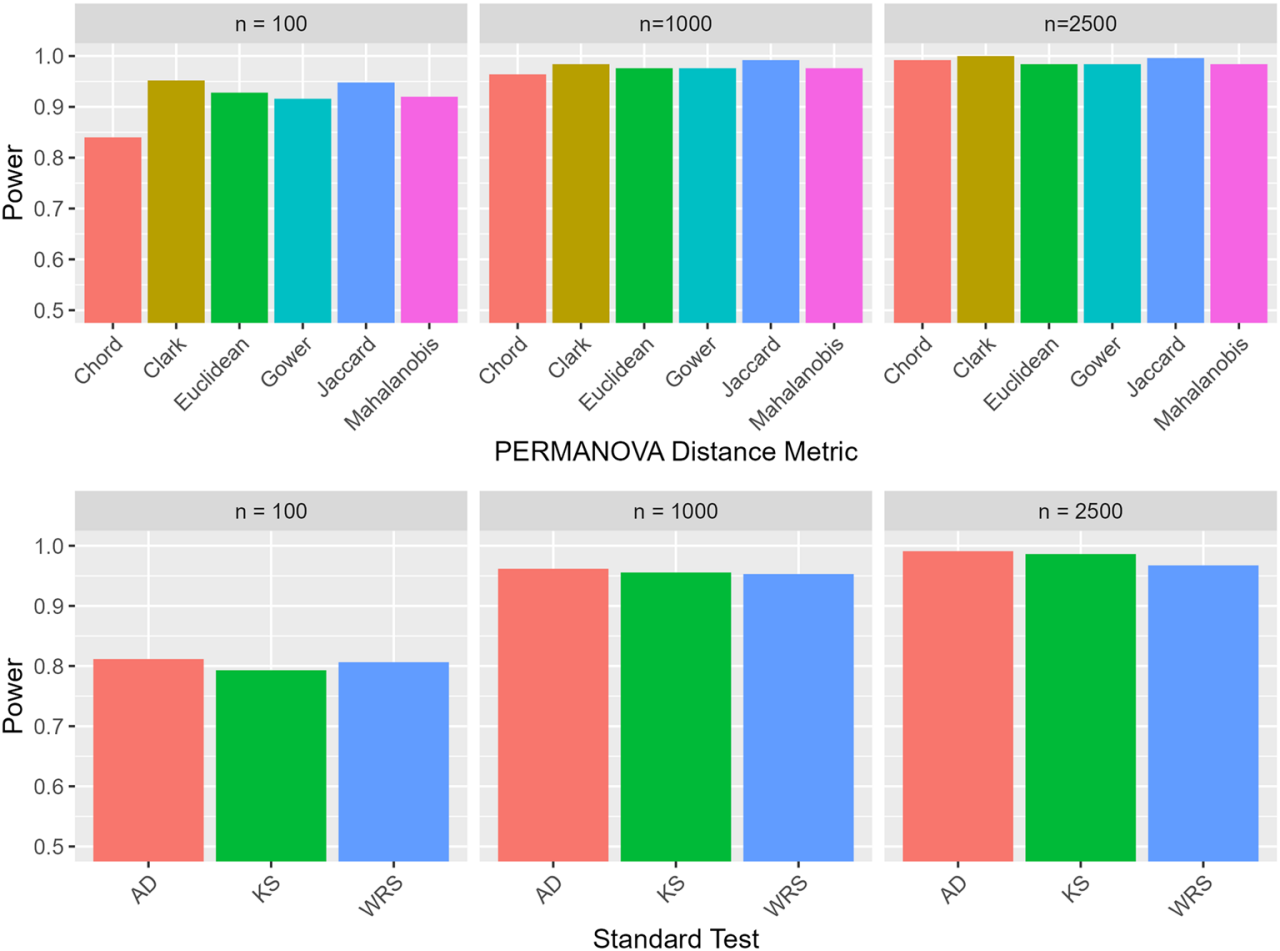
Power for using PERMANOVA and traditional feature-level statistical testing (Anderson-Darling, Kolmogorov-Smirnov, Wilcoxon Rank-Sum) to detect batch effects in simulated data at a significance level of 0.05. True negative rates were all approximately 0.95, aligning with the significance level.

In simulated data, PERMANOVA demonstrated higher power across all three sample sizes compared to standard univariate testing (Fig. 1). PERMANOVA with the Clark distance demonstrated the highest power at sample sizes of 100 and 2500 (0.952 and 1.0, respectively), while PERMANOVA with the Jaccard distance had the highest power at the sample size of 1000 (0.992). The Anderson-Darling test had the highest power and showed the best performance out of all the standard univariate tests (0.812, 0.962, 0.991 for $n=100, 1000, 2500$, respectively). For both PERMANOVA and standard univariate tests, Type 1 error rates were approximately at or under 0.05, indicating all tests were effectively controlling the Type 1 error rate at the specified significance level of 0.05. In addition, both methods exhibited increasing power with increasing sample size. An additional experiment was also conducted to compare standard testing performance on exponentially transformed features versus non-transformed features, and results indicate that the exponential transform had no effect of standard testing performance (Figure S1).

**Figure 2:**
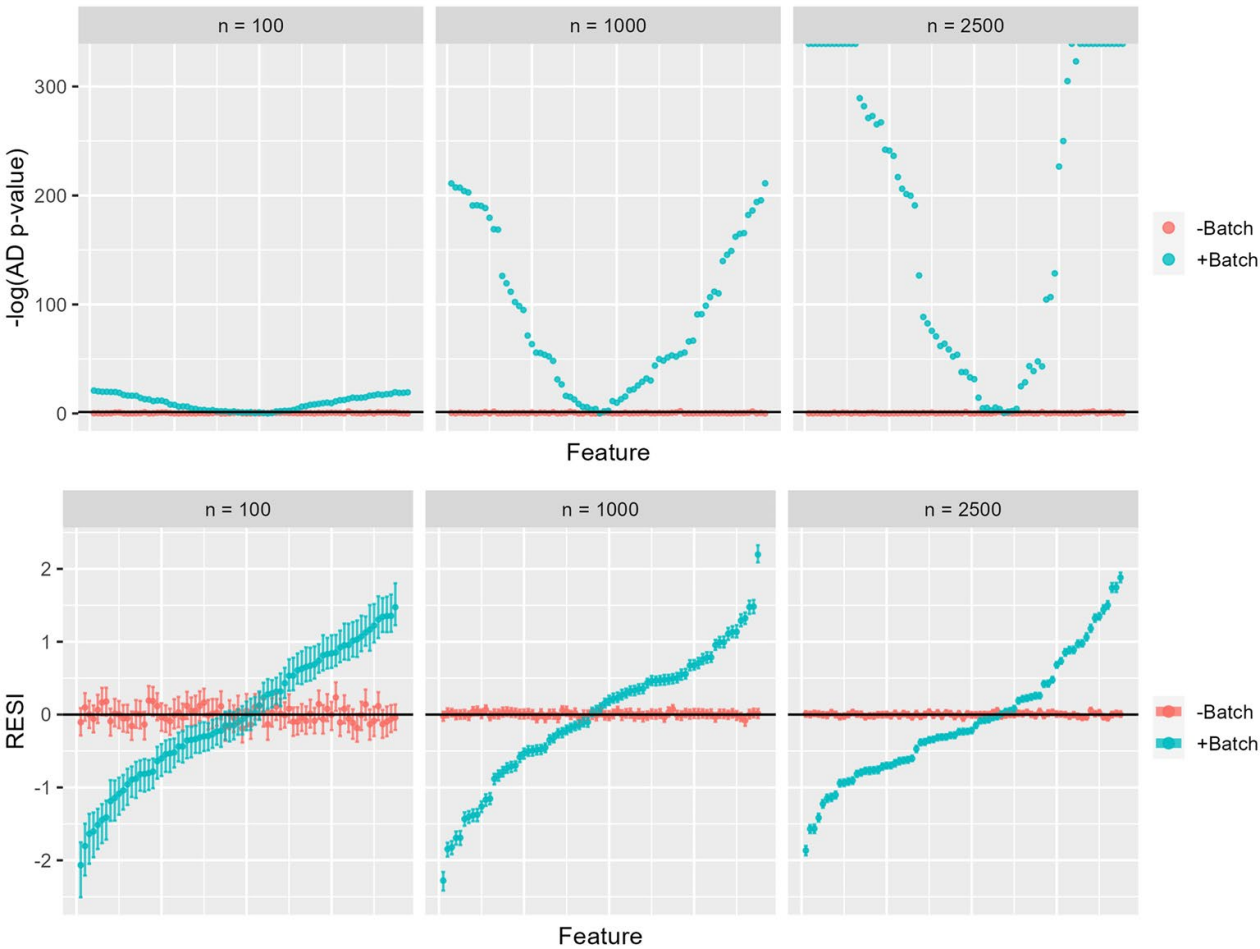
Anderson-Darling test p-values (on a negative log scale) and RESI for 75 randomly selected features from simulated data, sorted by increasing RESI. Red indicates no batch effects were added to the simulated data (-Batch), while blue indicates batch effects were added (+Batch). Values clustered at the top of the AD test plots indicate a p-value of 0, resulting in a plotted value of infinity. RESI error bars indicate the bootstrapped 95% confidence interval.

Results in simulated data for the RESI relative to the Anderson-Darling p-value are shown in Fig. 2. The AD test was selected for this comparison as the best performing out of all the standard univariate tests in simulated data and the only test applicable to the real radiomics dataset. The RESI was generally larger in magnitude when batch effects were added to the simulated data and corresponded to a smaller Anderson-Darling test p-value as well as rejection of the null hypothesis of no significant differences in distribution due to differences in simulated batch membership at a significance level of 0.05 (Table 1). In the absence of simulated batch effects, the RESI magnitude was closer to zero, which corresponded to larger Anderson-Darling p-values and acceptance of the null hypothesis for a significance level of 0.05. As sample size increased, the RESI estimates were more stable and demonstrated smaller 95% confidence interval width, indicating more precise estimates both with and without added batch effects (Fig. 2, Table 1). In contrast, the AD test p-value shrunk dramatically as sample size increased when simulated batch effects were added, in some features reaching a value of 0 when the sample size was 2500. In the absence of batch effects, Anderson-Darling test p-values still remained above the significance level of 0.05.

**Table 1:** Average absolute value of the RESI point estimate and 95% confidence interval width in simulated data. “+Batch” indicates simulated batch effects were added to the data, while “-Batch” indicates no batch effects were added.

-Batch		
	Point Estimate	95% CI Width
$n=100$	0.078	0.388
$n=1000$	0.025	0.123
$n=2500$	0.016	0.078

+Batch		
	Point Estimate	95% CI Width
$n=100$	0.683	0.455
$n=1000$	0.679	0.141
$n=2500$	0.685	0.090

### Radiomic data

**Figure 3:**
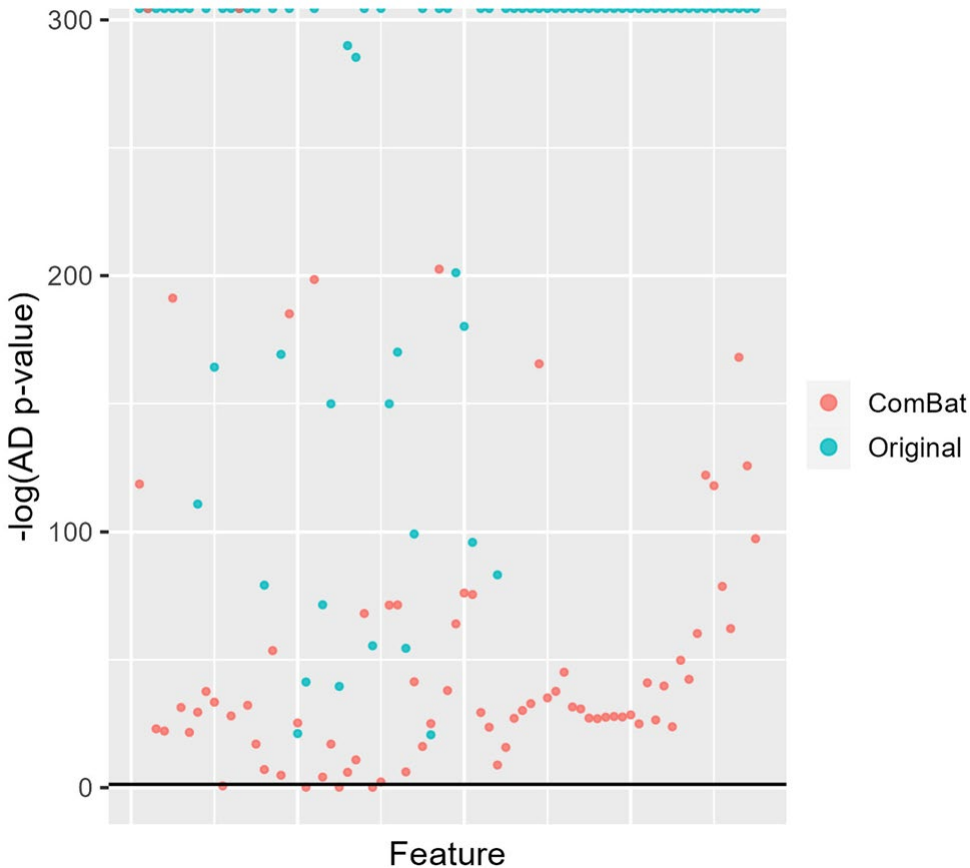
Anderson-Darling test p-values (on a negative log scale) for 75 randomly selected features from real radiomic data. Blue indicates the original unharmonized data (Original), while red indicates data that has been harmonized using ComBat. Values clustered at the top of the AD test plots indicate a p-value of 0, resulting in a plotted value of infinity. Black line in AD test plots is at the significance level ($\alpha$ = 0.05).

When the Anderson-Darling test was used to test for significant differences in distribution associated with center and study year combination in each feature at a significance level of 0.05, 99.6% and 95.7% of features had significant differences in distribution before and after ComBat harmonization, respectively. Anderson-Darling testing resulted in extremely small p-values for many features before harmonization, and after harmonization many features had p-values that were still below the significance level of 0.05 (Fig. 3). In addition, PERMANOVA testing yielded p-values < 0.05 across all distance metrics both before and after ComBat harmonization. PERMANOVA with the Mahalanobis distance was excluded from this analysis because small, near zero values in the covariance resulted in a singular, non-invertible matrix.

**Figure 4:**
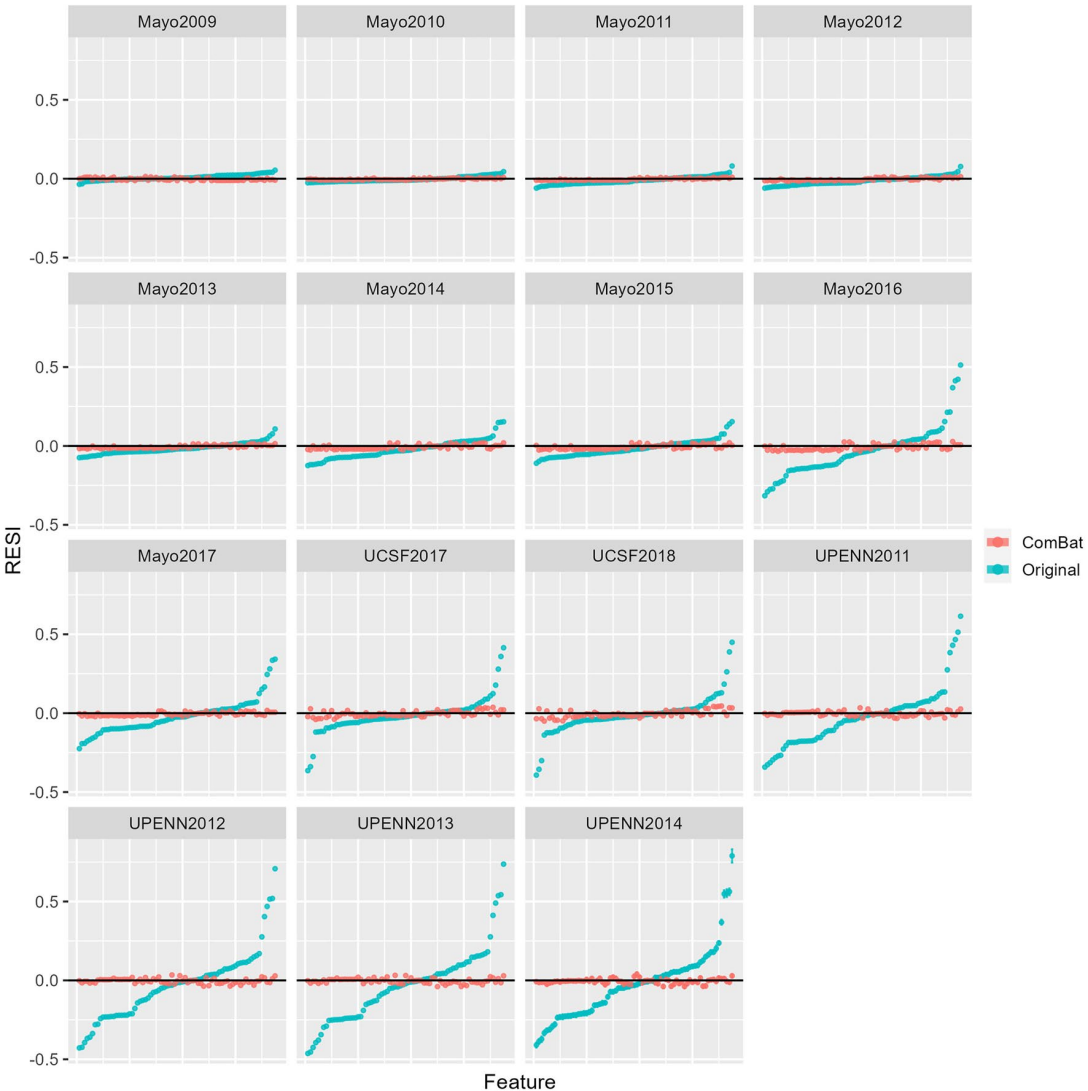
RESI for 75 randomly selected features from real radiomic data, sorted by increasing RESI. Blue indicates the original unharmonized data (Original), while red indicates data that has been harmonized using ComBat. Black line in the RESI plot is at 0, and RESI error bars indicate the bootstrapped 95% confidence interval. Because the 95% confidence intervals were very narrow, some error bars may not be visible. RESI was calculated for a linear model with batch as the categorical predictor and Mayo 2008 as the intercept such that each plot indicates the effect size for the remaining batch groups.

In contrast, calculating the point estimates for RESI for each feature resulted in RESI values that were larger in magnitude before harmonization and closer to zero after harmonization, indicating harmonization was successful in reducing the effect size of center/study year in the dataset (Fig. 4). It was also observed that given the reference level for the linear model used to calculate the RESI was Mayo 2008, the RESI in the original data increased with study year for the Mayo batch groups, indicating that the effect of center/study year increased over time. Effect sizes in the UPENN and UCSF batch groups also tended to be larger than in Mayo batch groups. The 95% confidence intervals were generally narrow, likely due to the large sample size.

## Discussion

While the radiomics community has used a wide range of traditional univariate statistical tests to detect significant differences in distribution due to batch effects, these tests can often exhibit reduced performance in settings where the image feature distributions are complex and non-Gaussian. In this work, we show that the PERMANOVA testing framework demonstrated the highest power and outperformed three traditional univariate tests in detecting batch effects in simulated data where it was known if batch effects have been added (Fig. 1). This improved performance was likely because PERMANOVA can detect complex nonlinear associations relationships without distributional assumptions and pooling information from the entire dataset. Also of note is that PERMANOVA with the Jaccard and Clark distances performed best, possibly because the applied exponential transform may be magnifying the batch effects. While in this study the Type 1 error rate remained below the significance level for all metrics, future work could assess whether magnifying these batch effects can result in over-sensitivity and increased Type 1 error.

Using a multivariate test that detects batch effects at the dataset-level as opposed to a feature-level test can enable more efficient screening of datasets to determine if harmonization is needed. The approach of drawing information from all features in the dataset using a multivariate test also more closely aligns with the ComBat assumption that batch effects affect features similarly, making PERMANOVA a more suitable metric for screening for batch effects prior to harmonization. In scenarios where a feature-level metric is needed for feature selection, the AD test performed best out of the standard univariate tests. The AD test likely performed better than the Wilcoxon Rank-Sum test because it tests for more generalized differences in distribution as opposed to location. In addition, as a k-sample test the AD test has more use cases than the KS test given that many imaging parameters that are candidates for harmonization contain more than two batch groups. While two-sample tests can be applied to k-samples by using a multi-comparison approach followed by post-hoc testing with p-value correction, this is not recommended because the p-value correction would result in reduced statistical power. Lowering the statistical power increases the risk of Type II error when detecting batch effects in data via statistical testing.

However, in extremely large datasets the p-values returned by standard univariate tests can shrink to extremely small values and give no information regarding the magnitude of the batch effect affecting the feature. AD p-values were essentially zero for several features in the simulated data with batch effects added at $n=2500$, and in the FFDM dataset before harmonization (Figs. 2, 3). In the FFDM dataset, the AD test detected significant differences in distribution due to center/study year in the majority of features both before and after harmonization, likely due to p-value shrinkage from the large sample size (Fig. 3). The findings from the AD test in this dataset are largely uninformative given increases in p-value cannot be interpreted as an decrease in the magnitude of the batch effect. Similarly, the PERMANOVA test indicated significant differences associated with center/study year in the dataset both before and after harmonization, also likely due to the large sample size (Table 2). While these small p-values suggest greater power to detect smaller effect sizes, if the p-value is below the significance level both before and after harmonization, it provides no information regarding harmonization performance.

These results indicate the need for effect size measures such as the RESI to quantify batch effects and better characterize harmonization performance in large datasets. P-values tend to decrease with increasing sample size because of reduced sampling error – while this makes the p-value less informative, this property improves the precision of an effect size metric like the RESI. The RESI was able to quantify the effect size of batch in features where the corresponding AD p-value was zero in both the simulated data and the FFDM data (Figs. 2, 3, 4). In the FFDM data, the RESI values were much closer to zero post-harmonization, showing that ComBat harmonization was effective in a dataset where the AD-test was uninformative (Fig. 4). It was also found that the effect size for center/study year increased with study year among the Mayo samples and that this effect size was larger in other centers (UPENN, UCSF). These observations indicated that images collected farther apart in time or at different centers differed more than images collected more closely in time or within the same center, demonstrating how RESI can be used to interpret differences in batch effects across groups. The ability to observe this phenomenon in the data also highlights that RESI can quantify the magnitude of the effect size for batch, as opposed to statistical testing that treats batch effects as a binary outcome. In addition, the RESI demonstrated narrower 95% confidence intervals and increased precision with larger sample size in both simulated and real data, as opposed to the AD test which became less informative in larger datasets. Analysis of feature families most affected by site and study year combination was also more informative when the RESI was used as opposed to the Anderson-Darling test, as results from the RESI were able to show that the neighbors feature family was most affected by batch before ComBat harmonization (Figures S2–S3). These results showed that the RESI is an interpretable measure that quantifies the effect size of batch in features that remains effective in large datasets.

In this work, we demonstrated that PERMANOVA is a more sensitive test than standard univariate tests for batch effect detection, and the Anderson-Darling test is the most sensitive standard univariate test. We also showed that the RESI can quantify the effect size of batch in individual features at large sample sizes where p-value shrinkage makes univariate testing uninformative, and can be used to measure harmonization performance in real radiomics data. These methods can be integrated into a single batch effect evaluation pipeline, where PERMANOVA is used to screen the data for batch effects at the dataset-level and the RESI is used to quantify the effect size of batch at the feature-level. Our findings are a promising initial study, and batch effect evaluation for high-dimensional imaging data remains an important and challenging open problem that requires further research. Future work includes additional simulations to assess the performance of PERMANOVA and RESI in study designs that result in more complex batch effects, including batch effects in the covariance structure, multiple batch variables, and confounding between batch and biological covariates of interest. Additional analyses with additional radiomic datasets extracted from real imaging data are needed to further validate our findings.

## Methods

We determine if PERMANOVA and RESI can be used as more powerful and interpretable metrics for batch effect evaluation by measuring their performance in detection of known batch effects in simulated data and characterization of harmonization effects in a radiomic dataset extracted from a multi-center mammography study. We also compare the performance of the proposed methods with more standard univariate statistical tests.

### Batch effect evaluation methods

Univariate statistical tests of the null hypothesis of no differences in distribution across batch groups are a common approach for detecting batch effects and assessing harmonization performance in radiomic datasets. We thus use these to benchmark batch effect detection performance relative to RESI and PERMANOVA^14,15,18^. In this work, we use the Wilcoxon Rank-Sum (WRS) test, the Kolmogorov-Smirnov (KS), and the Anderson-Darling (AD) test. All three tests have been used in batch effect detection for radiomics in literature^14,15,18^. Because simulations only included two batch groups, all three tests could be applied. In the real radiomic data more than two batch groups were present, so only the AD test could be applied.

The first method proposed to improve upon standard univariate statistical testing approaches for batch effect detection is PERMANOVA, which was first introduced by McArdle et al. as a distribution-free test for nonlinear associations in ecological data^26^. The test measures similarities between samples using a distance metric, partitions on known sources of variation, and approximates the permutational distribution to obtain a pseudo-F statistic comparing the within-group and between-group distances. This type of distance-based statistical testing has since grown in popularity as an effective test for high-dimensional data, with demonstrated applications in genomics and neuroimaging^27,28^. Moreover, its parametric counterpart Multivariate Analysis of Variance (MANOVA) has demonstrated applications in obtaining a dataset-level p-value for associations between site and features to assess harmonization in a neuroimaging dataset^19^. While a full derivation for PERMANOVA can be found in the original publication, a brief summary is as follows^26^:

Suppose an $n \times n$ matrix *D* is generated by calculating some distance metric over an $n \times m$ matrix of data *Y* for *n* samples and *m* features. This distance matrix can be used to compute the inner product matrix *A* with elements $a_{ij} = -\frac{1}{2}d_{ij}^2$ for row index *i* and column index *j*. The matrix *A* can be used to compute Gower’s centered matrix *G*, which is combined with the projection matrix *H* and $n \times g$ design matrix *X* to calculate the pseudo F-statistic for PERMANOVA:

1 $$\begin{aligned} F = \frac{tr(HGH)/(g-1)}{tr[(I-H)G(I-H)]/(n-g)} = \frac{SS_B}{SS_R} * \frac{n-g}{g-1} \end{aligned}$$

In this equation, the between group sum of squares $SS_B$ and the within group (residual) sum of squares $SS_W$ are computed using the traces of the described matrices. This F-statistic can then be tested with permutations, removing the assumption of normality held in standard ANOVA. In this work, we used the publicly available *adonis2* implementation of PERMANOVA in the *vegan* package in R. To determine if specific metrics were more suitable for radiomics, PERMANOVA was completed with several distance metrics (calculated using *vegdist* in the *vegan* package): Chord, Clark, Euclidean, Gower, Jaccard, and Mahalanobis. Because the Clark and Jaccard distances required positive feature values, an exponential transform was applied to the data prior to PERMANOVA. This transform was applied by taking the exponential of each entry of the original data matrix. In the simulated data, PERMANOVA was completed with 2000 permutations. The number of permutations was selected to minimize computational time while also obtaining robust estimates. Future studies could examine the effect of the number of permutations on the estimation quality.

The second proposed batch effect evaluation method is the RESI, which was developed by Vandekar et al. as a unitless measure of effect size that generalizes over a wide range of models to enable more standardized comparisons of effect size in the behavioral sciences^29^. The RESI is defined as the component of a chi-squared statistic that is associated with the deviation of a parameter from its reference value:

2 $$\begin{aligned} RESI = \sqrt{(\beta -\beta _0)^T\Sigma _\beta (\theta )^{-1}(\beta -\beta _0)} \end{aligned}$$

In this equation, $\Sigma _\beta (\theta )$ is the asymptotic covariance matrix for the estimate $\hat{\beta }$, and $\theta$ is the parameter that maximizes the value of the RESI estimating equation. A more detailed derivation of the RESI definition from an M-estimator and its corresponding estimator can be found in the original publication by Vandekar et al.^29^.

Kang et al. further improved upon RESI by creating a method for obtaining bootstrapped confidence and credible intervals to better characterize the uncertainty of the RESI estimate^30^. Both the RESI and its corresponding 95% confidence interval were calculated for each feature in the simulated data using a linear model with feature as the outcome and batch group as the predictor using the publicly available GitHub repository^31^. For faster computation, the number of bootstraps was reduced to $n=500$. The number of bootstraps was selected to minimize computational time while also maintaining estimation quality. Future studies could examine the effect of the number of permutations on the estimation quality.

### Simulation model

To assess batch effect detection performance in a scenario where batch effect status is known, simulations of radiomic features with added batch effects were simulated according to the following model:

3 $$\begin{aligned} Y_{ijg} = \gamma _i + \delta _i\varepsilon _{ijg} \end{aligned}$$

where *i* is the index for batch, *j* is the index for sample, and *g* is the index for feature. In this model, $\varepsilon _{i} \sim N(0, 1)$ are the simulated normally distributed residuals. We first multiplied these residuals by a simulated variance (scale) batch effect $\delta _i \sim Uniform (0.8, 1.2)$, then added a simulated mean (location) batch effect $\gamma _i \sim Uniform(-2, 2)$. Samples were randomly assigned to one of two batch groups for a single simulated batch variable. Simulations were completed with and without batch effects for 100, 1000, and 2500 samples for 250 iterations, with 20 features simulated per iteration. The number of features was selected to optimize computational efficiency given this is an initial study in the application of the proposed metrics for batch effect evaluation. Future simulation studies could include a higher number of features to better mimic real radiomic datasets, as both RESI and PERMANOVA can handle higher dimensional data. When batch effects were added to simulated features, they were applied to all features for the iteration. In this simulation, features were simulated with and without batch effects such that the batch effect status of a particular dataset is known, giving ground truth labels for each feature. Given that the ground truth is known in the simulated data, we computed the Type 1 error rate and power at the dataset-level for PERMANOVA (e.g. 20 features simulated during a single iteration is considered a single dataset) and at the feature-level for standard univariate tests. The Type 1 error rate and power were computed with respect to the null hypothesis that there were no differences in distribution across batch groups and the alternative hypothesis that such differences in distribution were present in the data. Both PERMANOVA and standard univariate testing were completed at significance level of $\alpha = 0.05$.

### Radiomic dataset

To assess the ability of these approaches to characterize batch effects in real data, we applied standard univariate testing, PERMANOVA, and RESI to radiomic features extracted from a large, multi-center full-field digital mammography (FFDM) dataset collected for the study of breast cancer risk^32^. A validated processing pipeline was used to automatically extract 1028 features from 29,321 FFDM scans collected at the Mayo Clinic, Rochester (Mayo), University of Pennsylvania (UPENN), and the University of California, San Francisco (UCSF)^33,34^. A summary table of the extracted features is shown in Table S2. The combination of center and study year was treated as the batch variable, with a total of 14 batch groups (Table 2). Additional imaging parameters for this dataset can be found in Table S1.

A harmonized dataset was generated by applying ComBat harmonization to correct batch effects associated with center/study year combination in the original data, while protecting the covariates age, average fibroglandular volume, and body mass index^12,13^. The harmonized dataset was used to assess the detection approaches’ ability to characterize the effects of harmonization. ComBat harmonization was selected because it has become an increasingly popular method for addressing batch effects associated with heterogeneity in image acquisition parameters in radiomic datasets, and batch effect detection methods are often employed to assess harmonization performance^10^. Applying the proposed detection approaches to real data enables evaluation of their performance in a common practical use case in radiomics. In addition, in real radiomics datasets the ground truth of whether batch effects are present in the data and to what extent is unknown. In this work we assumed that ComBat harmonization should at least partially correct batch effects in the data. We make this assumption because ComBat has demonstrated efficacy in multiple real datasets, and these metrics are intended only to look for differences across batch groups and not examine the cause^10,11^. Differences across batch groups can be caused by batch effects as well as confounding with biological effects, and identifying the cause of these differences can be the subject of future studies^35^. For both the original and harmonized data, the AD test and RESI were calculated at the feature-level and PERMANOVA was completed at the dataset-level. Only the AD test was used because the WRS and KS tests are restricted to comparing differences between two groups, when the radiomic dataset contained 14 batch groups.

**Table 2:** Distribution of center and study year batch groups for the FFDM dataset.

Center	Study year	Frequency
Mayo	2008	1075
Mayo	2009	1361
Mayo	2010	1183
Mayo	2011	1209
Mayo	2012	1201
Mayo	2013	1171
Mayo	2014	1156
Mayo	2015	1106
Mayo	2016	1219
Mayo	2017	375
UCSF	2017	1104
UCSF	2018	1801
UPENN	2011	1901
UPENN	2012	3507
UPENN	2013	4909
UPENN	2014	5043

### Supplementary Information

Supplementary Information.

## Data Availability

Simulated data are not included as the model used to generate the data is included in the manuscript. The multicenter mammography dataset analyzed during the current study are not publicly available due to the institutional agreement but are available from the corresponding author on reasonable request.
