# Supplementary material for: Multivariate testing and effect size measures for batch effect evaluation in radiomic features
Source: Sci Rep. 2024 Jun 17;14:13923. doi: 10.1038/s41598-024-64208-z (PMC11183083; doi:10.1038/s41598-024-64208-z)
Supplement: Supplementary file 1 — Supplementary Information. [file 41598_2024_64208_MOESM1_ESM.docx]

**Multivariate testing and effect size measures for batch effect evaluation in radiomic features**

**Hannah Horng^1,2,3*^, Christopher Scott^4^, Stacey Winham^4^, Matthew Jensen^4^, Lauren Pantalone^2^, Walter Mankowski^2^, Karla Kerlikowske^5^, Celine M. Vachon^4^, Despina Kontos^2,6+^, Russell T. Shinohara^2,3+^**

^1^ Department of Bioengineering, University of Pennsylvania, Philadelphia, PA, 19104, USA

^2^ Center for Biomedical Image Computing and Analysis (CBICA), Department of Radiology, University of Pennsylvania, Philadelphia, PA, 19104, USA

^3^ Penn Statistics in Imaging Endeavor (PennSIVE), Department of Biostatistics, Epidemiology, and Informatics, University of Pennsylvania, Philadelphia, PA, 19104, USA

^4^ Mayo Clinic, Rochester, MN, 55905, USA

^5^ University of California, San Francisco, CA, 94121, USA

^6^ Center for Innovation in Imaging Biomarkers and Integrated Diagnostics (CIMBID), Columbia University, New York, NY, 10027

*Corresponding author: [hannah.horng@gmail.com](mailto:hannah.horng@gmail.com)

^+^Co-senior authors

**SUPPLEMENTARY INFORMATION**

| Imaging Parameter | Value |
| --- | --- |
| Manufacturer | Hologic |
| Target Material | MOLYBDENUM, TUNGSTEN |
| Filter Material | MOLYBDENUM, RHODIUM, SILVER, MR, RS |
| Peak Kilovoltage | 0.03-0.057 kVp |
| Half Value Layer | 22-39 mm |

**Table S1**. Mammography acquisition parameters for the multicenter FFDM dataset.

| Feature | Count |
| --- | --- |
| Box Counting  Mean  Median  Standard Deviation | 1  1  1 |
| Cooccurrence  Mean  Median  Standard Deviation | 8  8  8 |
| Coocurrence Laws  Mean  Median  Standard Deviation | 120  120  120 |
| Edge Enhancement  Mean  Median  Standard Deviation | 1  1  1 |
| Gabor Wavelet  Mean  Median  Standard Deviation | 32  32  32 |
| Gray Level  Mean  Median  Standard Deviation | 12  12  12 |
| Laws  Mean  Median  Standard Deviation | 125  125  125 |
| Minkovski  Mean  Median  Standard Deviation | 1  1  1 |
| Neighbors  Mean  Median  Standard Deviation | 36  36  36 |
| Power Spectrum  Mean  Median  Standard Deviation | 1  1  1 |
| Run Length  Mean  Median  Standard Deviation | 5  5  7 |

**Table S2**. Table of extracted features for the multicenter FFDM dataset.


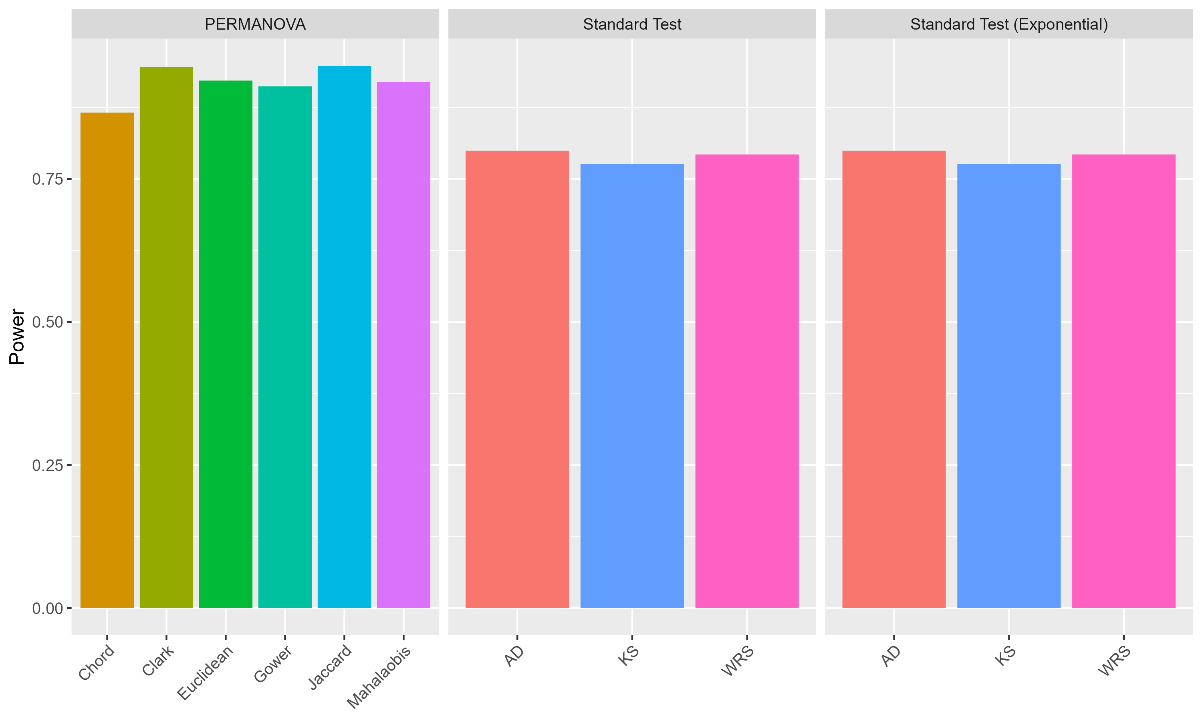


**Figure S1**. Power for using PERMANOVA and traditional feature-level statistical testing (Anderson-Darling, Kolmogorov-Smirnov, Wilcoxon Rank-Sum) to detect batch effects in simulated data at a significance level of 0.05, including feature-level testing on exponentially transformed features.

PERMANOVA with the Clark and Jaccard distance, as well as the standard test (exponential) results, were computed using exponentially transformed simulated features. All other results were computed using the standard features.


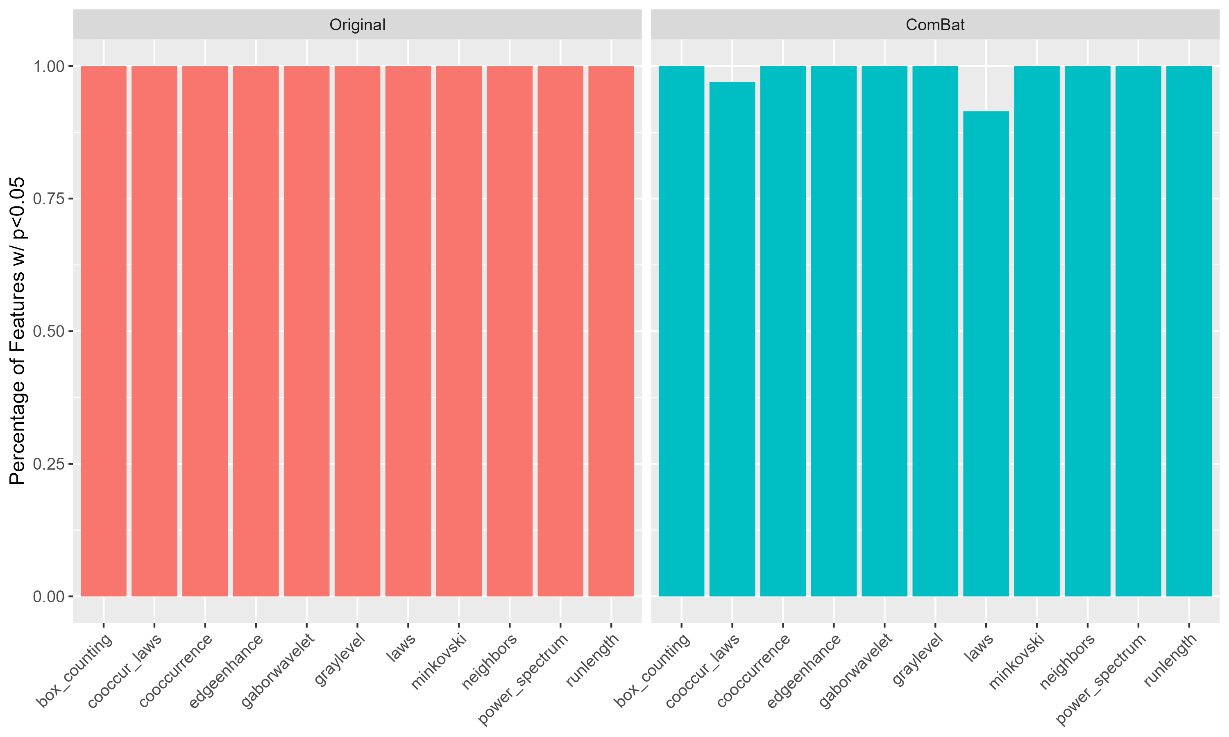


**Figure S2**. Percentage of features by feature family with statistically significant (p<0.05) differences in distribution attributable to site and study year combination detected by the Anderson-Darling test in the multicenter FFDM dataset.


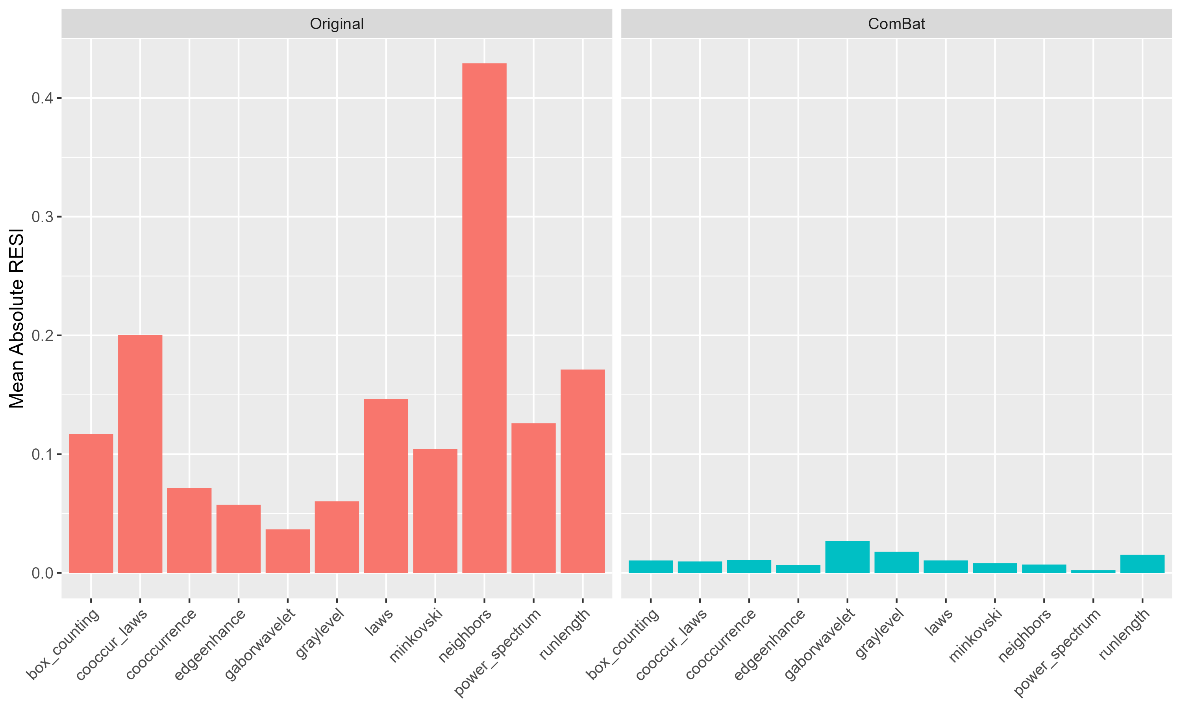


**Figure S3**. Mean of the absolute value of the RESI by feature family in the multicenter FFDM dataset.
